# Reticulocyte Binding Protein Homologue 5 is a target of balancing selection in the *Plasmodium falciparum* population of Papua New Guinea

**DOI:** 10.3389/fpara.2023.1288867

**Published:** 2023-12-22

**Authors:** Myo T. Naung, Elijah Martin, Wilson Wong, Zahra Razook, Digjaya Utama, Andrew J. Guy, Shannon Takala Harrison, Alan F. Cowman, Enmoore Lin, Benson Kiniboro, Moses Laman, Ivo Mueller, Alyssa E. Barry

**Affiliations:** ^1^ Population Health and Immunity Division, Walter and Eliza Hall Institute of Medical Research, Parkville, VIC, Australia; ^2^ Department of Medical Biology, University of Melbourne, Carlton, VIC, Australia; ^3^ Centre for Innovation in Infectious Diseases and Immunology Research (CIIDIR), Institute of Mental and Physical Health and Clinical Translation (IMPACT) and School of Medicine, Deakin University, Geelong, VIC, Australia; ^4^ Disease Elimination and Maternal and Child Health, Burnet Institute, Melbourne, VIC, Australia; ^5^ Bioscience and Food Technology, RMIT University, Melbourne, VIC, Australia; ^6^ Center for Vaccine Development and Global Health, University of Maryland School of Medicine, Baltimore, MD, United States; ^7^ Vector Borne Diseases Unit, Papua New Guinea Institute of Medical Research, Madang, Papua New Guinea; ^8^ Parasites and Insect Vectors, Pasteur Institute, Paris, France

**Keywords:** *Plasmodium falciparum*, RH5, vaccine, polymorphisms, haplotypes, immune escape, malaria

## Abstract

*Plasmodium falciparum* Reticulocyte Binding Protein Homologue (RH5), a leading malaria vaccine candidate, is essential for erythrocyte invasion by the parasite, interacting with the human host receptor, basigin. RH5 has a small number of polymorphisms relative to other blood-stage antigens, and *in vitro* studies have shown that vaccine-induced antibodies raised against RH5 are strain-transcending, however most studies investigating RH5 diversity have been done in Africa. Understanding the genetic diversity and evolution of malaria antigens in other regions is important for their validation as vaccine candidates. In this study the *rh5* gene was sequenced in 677 samples from a longitudinal cohort of Papua New Guinean (PNG) children aged 1-3 years. Of 677 samples successfully sequenced, 566 were identified as independent infections (i.e. one of each pair of identical sequences within hosts were removed). A total of 14 non-synonymous polymorphisms were identified, eight that are ‘common’ in the population (minor allele frequency > 1%), with 44 haplotypes ranging in frequency from 1% to 21%. Modeling of common SNPs to the cryo-EM structure of the RH5/CyRPA/RIPR complex mapped them to the Basigin binding site and near the contact point of CyRPA. Tajima’s *D* analyses of the corresponding nucleotide sequences produced positive values indicating potential hotspots of balancing selection. We attempted to confirm whether these signals were due to immune selection by measuring the rate of polymorphism between independent infections within the same host, and the association with clinical symptoms, however, no such associations were identified. Together these results suggest that while there is evidence of balancing selection driving RH5 diversity in the PNG *P. falciparum* population, immune escape was not observed within the cohort of young children. Limited immunity and therefore low selective pressure may explain this result, alternatively other evolutionary forces may contribute to balancing selection at the RH5-BSG binding interface in PNG.

## 1 Introduction

Despite recent progress in reducing the burden of malaria, the disease remains a major global health problem, with 619,000 deaths and 247 million clinical cases in 2021 heightened by the spread of antimalarial drug and insecticide resistance and widespread COVID-19 mitigation measures interrupting malaria services (World Health Organization (WHO), 2022). As a result, more sustainable strategies to control and reduce the disease burden, such as a broadly effective malaria vaccine, are needed to accomplish global malaria eradication (World Health Organization (WHO), 2022). Developing malaria vaccines with long-lasting efficacy is an enduring challenge due to the extensive genetic diversity of parasite surface antigens used in so-called ‘subunit’ vaccines (Barry et al., 2009; Takala et al., 2009). Much of this diversity is thought to have evolved as a means for parasites to evade the host immune response (Gupta et al., 1998; Weedall and Conway, 2010) and therefore these antigens only protect against parasites carrying the same or similar alleles. To achieve broad efficacy against diverse strains, malaria vaccines may need to include multiple alleles.

In endemic areas, people are repeatedly infected with malaria parasites, however, immunity is eventually acquired to disease symptoms (Day and Marsh, 1991) and is associated, in part, with the accumulation of a diverse repertoire of immune responses to different strains (Doolan et al., 2009). Allele-specific immune responses have been observed against specific parasite antigens suggesting that parasites carrying different alleles are responsible for repeated infections, and if vaccines are formulated with a single allele, parasites carrying alternative alleles may escape the induced immune response and therefore predominate in breakthrough infections following vaccination (Takala et al., 2007; Thera et al., 2011; Ouattara et al., 2013; RTS, S Clinical Trials Partnership, 2015; Shah et al., 2021). The most advanced malaria vaccine candidate, RTS, S/AS01, formulated with the 3D7 allele only, has greater efficacy against parasites with alleles matching the vaccine strains compared to diverse alleles in the population (Neafsey et al., 2015; RTS, S Clinical Trials Partnership, 2015; Beeson et al., 2019; Laurens, 2019). Moreover, if the vaccine allele is at low frequency in the population, immune escape could limit overall protective efficacy (Takala et al., 2009; Barry and Arnott, 2014). Knowledge of the diversity of vaccine antigens, and more specifically, polymorphisms that contribute to immune escape can aid the selection of representative alleles for inclusion in a multivalent vaccine (Quiñones-Parra et al., 2014; Ouattara et al., 2015; Han et al., 2021).

*Plasmodium falciparum* reticulocyte homolog 5 protein (RH5), a 63 kDa protein encoded by a gene (PF3D7_0424100), forms a pentameric complex with CyRPA, RIPR, and TRAMP (called PCRCR complex) after releasing from the rhoptries (Wright et al., 2014; Wong et al., 2019; Scally et al., 2022). This facilitates RH5 expression on the merozoite surface during the invasion phase. The interaction of RH5 with the receptor basigin (CD147) through hydrogen bonds is essential for merozoite invasion of erythrocytes (Cowman and Crabb, 2006; Baum et al., 2009; Crosnier et al., 2011; Wright et al., 2014; Galaway et al., 2017). Being an invasion ligand, RH5 is a target of inhibitory antibodies that prevent parasite invasion and replication within red blood cells (RBCs) (Cowman and Crabb, 2006; Wright et al., 2014). Both *in vitro* and *in vivo* studies showed that antibodies that target RH5 can inhibit *P. falciparum* erythrocytic invasion, in a dose-dependent manner (Crosnier et al., 2011; Tran et al., 2014; Wright et al., 2014). Due to its essential role in invasion, RH5 has been prioritized as a new blood stage vaccine antigen and is approaching clinical development (Douglas et al., 2015; Campeotto et al., 2017).

Unlike other leading malaria subunit vaccine candidates such as apical membrane antigen-1 (AMA1) and merozoite protein-1 (MSP1), RH5 is considered to be conserved with limited polymorphism (Ndwiga et al., 2021) and polyclonal IgG antibodies raised against it have strain transcending activity in *in vitro* growth inhibition assays (Douglas et al., 2011). Of the 11 non-synonymous polymorphisms identified globally, eight are considered ‘common’ (>1% frequency) (Naung et al., 2022) but previous investigations did not identify any evidence of immune selection (Ouattara et al., 2018). Nevertheless, RH5 gene sequences from African isolates were recently shown to be more diverse than previously described (Mangou et al., 2022). However, analysis of RH5 gene sequences in parasite populations in the Asia-Pacific suggest that diversity may be driven by balancing selection (Naung et al., 2022). Given the complexity of host-parasite interactions and selective forces in different settings, further investigation of the contribution of RH5 polymorphism to immune escape in Asia-Pacific parasite populations is warranted.

In naturally infected human populations, each *P. falciparum* infection results in the acquisition of strain specific immune responses, which are associated with a reduction in the prevalence of clinical infections with repeated infections (Ouattara et al., 2013). If immune selection is a driver of RH5 diversity, *rh5* gene alleles will be present at intermediate frequencies resulting in a signal of balancing selection. This study aimed to investigate RH5 gene diversity in a longitudinal cohort of Papua New Guinean (PNG) children with high rates of natural malaria exposure. The extent of polymorphism, diversity and balancing selection was measured using population genetic analyses. Diversity was mapped to 3-dimensional protein structures to explore potential biological roles of polymorphism. In addition, there should be a higher rate of allelic change between infections within the same host than if two randomly selected pairs of infections from different hosts are compared. We thus evaluated allelic turnover between infections as a measure of immune selection within individual hosts. The results provide novel findings into the relevance of RH5 diversity for malaria vaccine development.

## 2 Methods

### 2.1 Samples

Samples comprised archived parasite isolates from a longitudinal cohort of children living in villages surrounding Ilaita, East Sepik Province, PNG during a period of high transmission (Lin et al., 2010; Mueller et al., 2012). A total of 264 children aged 0.9–3.2 y (median, 1.7 y; interquartile range, 1.3 - 2.4 y) were enrolled between March and September 2006 and were followed for up to 69 weeks. The majority (93.9%) of participants were sampled every 8 weeks for the entirety of the study (Lin et al., 2010; Mueller et al., 2012). Previous clinical and parasitological measures classify infections as asymptomatic and symptomatic. This cohort was chosen because this age group in PNG is partially immune and therefore actively acquiring immunity to malaria. As a result, these young children experienced numerous clinical episodes during the course of the study. Symptomatic malaria cases were defined as blood stage *P. falciparum* parasitemia of greater than or equal to 2500 parasites/*µ*l by PCR-ligase detection reaction (LDR) with fever (auxiliary temperature > 37.5 C) within 48 h of sampling. Asymptomatic malaria cases were defined as *P. falciparum* greater than or equal to 2500 per *µ*l, positive light microscopy similar to the threshold used in previous analysis (Tessema et al., 2019), and positive PCR-ligase detection reaction (LDR) without fever in preceding 48 h. Individuals with malaria onset symptoms following 48 h period were captured via passive detection (presentation to the clinic with malaria symptoms). In preparation for sequencing, all available symptomatic malaria infections and up to three asymptomatic infections per individual were included, resulting in 1475 P*. falciparum* isolates available for the study.

Ethical approval for the study was provided by the Medical Research Advisory Council of PNG No. 07/11, the Walter and Eliza Hall Institute Human Research Ethics Committee No. 07/07, and the Deakin University Human Research Ethics Committee No. 2023-219.

### 2.2 PCR and sequencing

Genomic DNA extraction was done and *P. falciparum* infections identified by quantitative PCR as detailed in Lin et al. (2010) (Lin et al., 2010). Nested PCR was used to amplify nucleotide positions 292-1709 of the full-length *rh5* gene [Genbank Accession Number: NC_004318.2]. The protocol included two rounds of amplification with amplicons from the first reaction as templates for second PCR step with the following PCR primers (Integrated DNA Technologies, Australia): external forward, 5’- ACAATGATAATTGTGTCATC -3’; external reverse, 5’-ACAAATGAAGTTCAATGATGTCCCA -3’; internal forward, 5’ -TGTACAGGATTAAGTTTTGAAAATGCA -3’; and internal reverse, 5’- AATAAACCACTTACACAATG -3’. Each 25 *µ*l PCR reaction consisted of 2.5 *µ*l of 10x buffer, 2.0 *µ*l of 50mM MgCl_2_ (Solis Biodyne), 2.0 *µ*l l of 25 mM deoxynucleotide triphosphate (dNTPs) (Solis Biodyne), 1.0 *µ*l of each 15 pmol of each forward and reverse primers, 0.3 *µ*l l of *Taq* polymerase (Solis Biodyne), 15.2 *µ*l of HPLC gradient grade water, and 1 *µ*l of genomic template DNA. The same thermocycling parameters were used for all PCR reactions: initialization for 12 mins at 95 C followed by 35 cycles of denaturation at 94 C for 30 s, annealing at 55 C for 30 s, extension at 68 C for 1.5 min, and one cycle for the final extension at 68 C for 7 min with a hold at 4 C. 3D7 template DNA (positive control) and no template control (negative control) were included in all PCR assays to detect contamination and unintended amplification products. PCR products were visualized using 1% agarose gel electrophoresis.

Forward and reverse sequencing of *rh5* amplicons was performed by a contract sequencing facility (Macrogen, Seoul, Korea) using Sanger sequencing with the ABI BigDye Terminator Cycle Sequencing kit on an ABI 3730XL automatic DNA Analyzer. Chromatograms were processed as per previously described methods (Arnott et al., 2014) in Geneious software (version 6.1.8). Editing of chromatograms included trimming the lower-quality ends of the sequenced reads and mapping them to the reference 3D7 *rh5* sequence (PF3D7_0424100). Once mapped to the reference, ambiguous base calls were clarified by visually inspecting chromatograms. Low quality or truncated sequences, as defined by software-defined default thresholds were removed from the analysis. Infections were classified as multiple infections if heterozygous positions were observed within the chromatograms, where only the dominant allele was called. Dominant alleles were defined automatically by the software where the minor peak was below 30% relative to the primary peak however were called manually in cases where the minor peak was equal to or greater than 30%. High quality consensus sequences were obtained by merging forward and reverse reads that passed the quality control filters.

### 2.3 Population genetic analyses

High quality consensus sequences for each isolate were aligned using the MUSCLE algorithm (Edgar, 2004) implemented in MEGA software (version 7.0) (Kumar et al., 2016) using default parameters. The co-ordinates of SNPs were identified based on the entire coding region of the *rh5* gene based on 3D7 coordinates (1581 base pairs). As they might represent PCR or sequencing artefacts, singleton SNPs were removed. These were defined as minor alleles found only once among all isolates, while SNPs present in at least one other isolate were considered independently confirmed and therefore retained. Haplotypes were defined as a given combination of polymorphisms without a particular weight on specific or functionally important polymorphic residues. Repeated haplotypes found in consecutive infections of the same individual host were only included once in the population genetic analysis such as nucleotide diversity, haplotype diversity, *Tajima’s D* statistics, and haplotype network as they may be the same parasite clone.

Based on multiple alignment of the new PNG sequences only, the number of total polymorphic sites (S), synonymous (SP), and non-synonymous (NS) single nucleotide polymorphisms (SNPs), Nei’s average pairwise nucleotide diversity (π)^36^ (Nei, 1987), number of amino acid haplotypes (h), and amino acid haplotype diversity (Hd) were calculated. These calculations were implemented in a custom R software package, *vaxpack*, available at https://github.com/BarryLab01/vaxpack. Relationships among *rh5* haplotypes based on non-synonymous polymorphisms were determined by constructing genetic networks using the Templeton, Crandall, and Sing (TCS) method in *PopArt* (version 1.7) (Leigh and Bryant, 2015).

To assess if *rh5* is under selection in context with the arrangement of amino acids within the 3-dimensional protein structure, we measured Tajima’s *D* (Tajima, 1989) using the gene sequences and incorporated spatial coordinates for the available Cryo-EM RH5 structure (PDB code: 6MPV) (Guy et al., 2018; Wong et al., 2019). The modified spatial Tajima’s *D* analysis differs from standard Tajima’s *D*, which is calculated based on the linear sequence. This spatially derived *Tajima’s D* analysis was done with a sliding window of 15A° using BioStructMap Python package (Guy et al., 2018) (available at https://github.com/andrewguy/biostructmap). The output from BioStructMap was visualized with Chimera X (Pettersen et al., 2004).

### 2.4 Measuring immune escape within hosts

To assess if specific polymorphic positions are associated with immune escape, we investigated the turnover of *rh5* alleles between consecutive infection pairs within hosts (**Supplementary Figure 1**). With the assumption that immune escape results in clinical symptoms, we focused the analysis on infection pairs from the same host, where the second infection was symptomatic (i.e., symptomatic to symptomatic, or asymptomatic to symptomatic transitions). The ‘pairwise mismatch score’ was calculated for each SNP using a novel algorithm, scoring 1 for mismatches and 0 for matches, by comparing sequences from all possible infection pairs (within and between host) (**Supplementary Figure 1**). An ‘expected’ score was determined by comparing randomly chosen infection pairs from different individuals in the entire cohort sequence database. The expected mismatch score was considered a measure of the background allelic change rate influenced by the observed allele frequencies in the entire parasite population. In cases where *rh5* polymorphisms are associated with immune escape, a significantly higher mismatch score is expected within-hosts compared to between hosts infections. For statistical testing of the significance, a permutation test with 999 randomized pairwise comparisons was used to calculate the p-value. The analysis was conducted using R (version 4.0.0). Raw data are available at https://github.com/myonaung/RH5_et_al.

## 3 Results

### 3.1 Data summary

A total of 1021 samples (*P. falciparum* isolates) from 264 individuals were selected for *rh5* gene PCR and sequencing. Of these, 677 (66%) produced high-quality *rh5* sequences (GenBank Acc No. MT414033-MT414709.). ‘Heterozygous’ positions where two or more SNP alleles were detected, occurred in 11% (75) of these sequences, indicating mixed-clone infections, where the dominant alleles were used to define haplotypes. To accommodate population genetic analyses which needs to exclude repeated sampling of the same clone within individuals, 111 identical sequences from consecutive infections were removed from the within-host sampling, leaving 566 sequences for the population level analyses. For within-host analyses which required paired samples, we conducted the analysis with these pairs. At least one pair of sequences was obtained for 170 of 264 individuals in the cohort study resulting in a total of 633 sequences (after removal of individuals for which only one sequence was available) for within-host analyses.

### 3.2 Polymorphisms and diversity

Based on the analysis of polymorphisms in 566 *rh5* sequences, fourteen non-synonymous and one synonymous polymorphism (1323 A/G) were identified (**Table 1**). The resulting amino acid polymorphisms include E50K, Y147H, H148D, S197Y, C203Y, A233E, N293K, M304R, D305E, N320T, V371I, S381L, I410M, K429N (**Supplementary Figure 2**). Eight of these polymorphic residues have minor allele frequencies greater than 1%. The most common polymorphisms were K429N (36.41%) followed by H148D (22.61%; **Supplementary Figure 2**). E50K, A233E, N293K, M304R, D305E and N320T were rare being found in less than 1% of the population (**Supplementary Figure 2**).

**Table 1:** Rh5 gene diversity in PNG *P. falciparum* isolates.

Clinical Status	n	Length	S	SNP	π x 10^-3^	D	NS	SP	h	Hd
Asymptomatic	291	1581	11	11	1.455	-0.128	14	1	35	0.900
Symptomatic	272	1581	15	15	1.397	0.583	11	0	27	0.896
Total	563	1581	15	15	1.427	0.090	14	1	44	0.898

n, number of sequences; length, nucleotide base pair; S, number of segregating (polymorphic) sites; π x 10^-3^, nucleotide diversity (Nei’s method); D, overall Tajima’s D; NS, non-synonymous single nucleotide polymorphisms; SP, synonymous single nucleotide polymorphisms; h, number of unique amino acid haplotypes; Hd, haplotype diversity or heterozygosity. Haplotype diversity (Hd) or heterozygosity is calculated as Hd = [n/(n - 1)] [1 - Σ (f_i_)^2^], where n is the sample size, and f is the frequency of the i^th^ allele.

**Figure 1:**
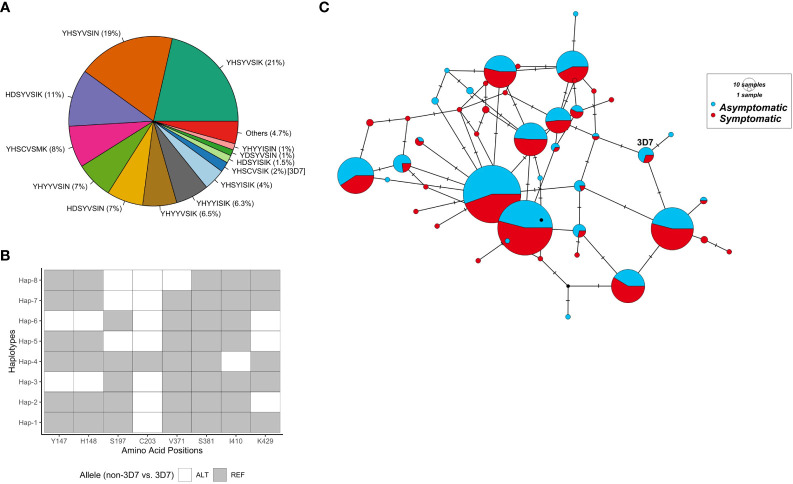
RH5 haplotypes in the PNG *P. falciparum* population. **(A)** RH5 haplotypes and frequency. Each haplotype in the pie chart is labelled with haplotypes based on amino acid residues at eight polymorphic positions - 147, 148, 197, 203, 371, 381, 410, and 429. **(B)** Common RH5 haplotypes (> 5%) in the PNG P. falciparum population and relationships to the 3D7 *P. falciparum* reference. Haplotypes are listed in the ascending order of frequency. The two most common haplotypes (Hap-1 and Hap-2) differ only at amino acid position 429. **(C)** Haplotype network showing rh5 haplotype relationships. Circles represent unique haplotypes, and are scaled according to prevalence. The number of non-synonymous SNP differences between each haplotype was shown by the number of hatch marks on the branches. The reference strain 3D7 was included for reference. Abbreviations for the amino acid residues are as follows: D, Asp; E, Glu; F, Phe; G, Gly; H, His; I, Ile; K, Lys; L, Leu; M, Met; N, Asn; P, Pro; Q, Gln; R, Arg; S, Ser; and Y, Tyr.

Based on the 14 non-synonymous polymorphisms, 44 unique haplotypes were identified within the PNG population. Twenty of the 44 haplotypes were rare with prevalence of less than 5%. Considering only the eight ‘common’ polymorphisms (more than X% prevalence), 30 unique haplotypes were found (**Figure 1A**). The 3D7 haplotype (YHSCVSIK) was present but was relatively rare (2%) being the tenth most common haplotype (**Figure 1A**). The dominant haplotype (Hap-1: YHSYVSIK, 21%) had only one amino difference with the 3D7 haplotype, at amino acid position 203 but this was located within the basigin binding site suggesting it may have a significant impact on antibody or receptor binding. The next most prevalent haplotype (Hap-2: YHSYVSIN, 19%) was present at a prevalence of 19%, with two differences compared to 3D7 at residues 203 and 429 (**Figures 1A, B**). As a result of the large number of haplotypes, the nucleotide and haplotype diversity were high (**Table 1**).

Interesting findings were observed when the data was stratified by clinical malaria status of the host at the time of infection. The S381L polymorphism was found only within symptomatic infections compared to a minor allele frequency of 2% overall, and thus this polymorphism may be associated with virulence. Nevertheless, overall diversity and the distribution of common haplotypes were similar between symptomatic and asymptomatic infections (**Table 1**; **Figure 1C**). We found that the less common haplotypes connected the major haplotypes suggesting they are recombinants of major haplotypes. Haplotypes specific to symptomatic infections were also found (due to the S381L polymorphism) but these were rare in the overall population (2%). In addition, low frequency haplotypes (n = 19) were found mostly within symptomatic infections (**Figure 1C**).

### 3.2 Natural selection

**Figure 2:**
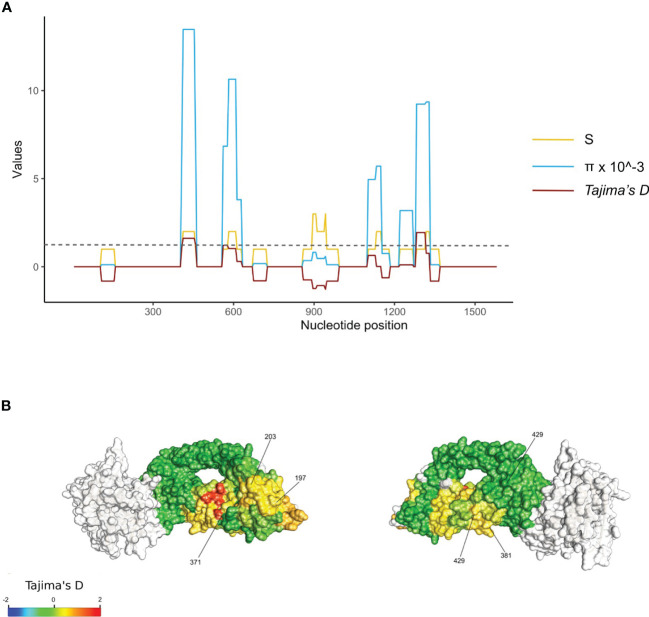
Balancing selection of the rh5 gene in the PNG *P. falciparum* population. **(A)**. Diversity of the rh5 gene. Sliding windows of the linear nucleotide sequence (window size of 50 bp and a step size of 5 bp) were calculated for segregating sites (S, yellow lines), nucleotide diversity (π, blue lines), and Tajima's D (D, red lines) for sequences obtained from all isolates (n = 667). The results were plotted together and scaled to the Tajima's D values. The grey dotted line indicates significant values for Tajima's D at p-value 0.1 for a respective sample size. Nucleotide positions are shown on the x-axis of each plot. **(B)** Balancing selection mapped to RH5 3 dimensional structure. Spatially derived Tajima's D scores were mapped into the Cyro-EM structure of RH5 (PDB code: 6MPV.B) for total isolates (n = 667). Tajima's D was calculated using a 3D sliding window with a radius of 15 Å for each window using BioStructMap Python package^33^. The structure was colored according to D scores mapped to each residue with undefined D shown in grey. The CyRPA-RIPR complex was also indicated in grey. Key polymorphic sites were labeled.

*Tajima’s D* values were mapped to the 3-dimensional protein structure. Polymorphic residues were all found on the surface of RH5 protein, with some found to cluster together in 3D space (**Figure 2A**). High focal Tajima’s *D* scores (1.05 – 1.76 by spatial *Tajima’s D* or 1.03 – 2.12 by linear *Tajima’s D*) were observed at residues L188 – F209, N338 – N347, S370 – D382, and F421 - K441, which contained polymorphisms S197Y and C203Y and overlap with the basigin binding site (**Figure 2**). The strongest *D* scores were found at polymorphic residue V371I using spatial *Tajima’s D* analysis or K429N using traditional linear level *Tajima’s D* analysis. In the linear sliding window analysis, a high *Tajima’s D* of 1.9 was also observed at amino acid residues Y147 and H148 (nucleotide positions 439 to 444, in the 3D7 sequence) near the N-terminal intrinsically disordered region, which were not included in the 3D structure (**Figure 2B**).

### 3.3 Within-host dynamics

Under immune escape, it is expected that the rate of non-synonymous substitutions between infections within the same host will be higher those found between any two infections between hosts (i.e. in the overall parasite population). It is also expected that infection with parasites carrying antigenically distinct variants would result in malaria symptoms in a subsequent infection. To measure this association, we therefore compared *rh5* sequences labeled as symptomatic to a previous infection (either asymptomatic or symptomatic). The dataset included 303 asymptomatic to symptomatic infection pairs and 268 symptomatic to symptomatic infection pairs for which we calculated total mismatch scores to evaluate whether any of the 14 non-synonymous *rh5* polymorphisms were associated with immune escape. As a negative control, we also included the synonymous mutation at nucleotide position 1323 (amino acid position 442).

**Figure 3:**
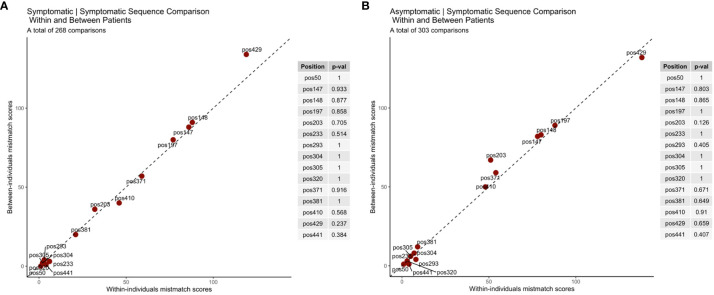
RH5 polymorphisms associated with immune escape. Comparison of sequences between **(A)** symptomatic to symptomatic and **(B)** asymptomatic to symptomatic infection pairs. Under immune selection rh5 sequences from the same individual are expected to be antigenically distinct alleles. Immune selection would therefore result in higher rates of allelic change (mismatch scores) for within infection pairs compared to randomly selected infection pairs from different hosts (i.e. the ‘background’ mutation rate). The diagonal line indicates the value if polymorphic scores within and between hosts are equal. P-values for each amino acid position indicate the results of the permutation tests.

The analysis demonstrates that the within-host polymorphism mismatch score and the between-hosts mismatch score were similar (**Figure 3**). This suggests that in this cohort of young children, the turnover of *rh5* alleles within-hosts is similar to the background change rate in the parasite population as a whole, and thus was not associated with clinical symptoms of individual hosts. This included SNPs at nucleotides 590 (S197Y) and 608 (C203Y), which are located at the basigin binding site, and under balancing selection at the parasite population level.

## 4 Discussion

Diversity in parasite antigens is a barrier to the development of effective malaria vaccines. Low diversity in RH5, an essential ligand for *P. falciparum* invasion of human red blood cells, has been used as a rationale to further develop this antigen as a malaria vaccine candidate (Douglas et al., 2011; Naung et al., 2022). Investigations of *rh5* gene diversity in whole genome sequence data from PNG compared to other populations indicates that despite there being few polymorphisms, this antigen may be under balancing selection, which could indicate selective pressure from host immune responses that would undermine vaccine efficacy (Naung et al., 2022). With eight common non-synonymous polymorphisms (minor allele frequency >1%) and high haplotype diversity (Hd=0.90) in this population sample from PNG, our data suggest that *rh5* is more diverse than previously recognized with polymorphisms C203Y and S197Y at a prevalence of 89% and 22%, respectively. The dominance of the tyrosine residue at position 203 of the protein, which is the alternative allele compared to the reference sequence 3D7, is aligned with previous reports from Malian children, and the ‘global’ MalariaGEN dataset (Ouattara et al., 2018; Galaway et al., 2019). Polymorphisms Y147H, H148D, S197, C203Y, V371I, and K429N correspond with the regions of the gene under significant balancing selection and include residues at the basigin binding site (S197Y and C203Y). However, the balancing selection is not as strong as that in other parasite surface antigens as AMA1 (Escalante et al., 2001; Takala et al., 2009; Naung et al., 2022). Residues S197Y and C203Y are physically proximal to each other and thus may be contained within the same epitope. These findings support previous *in vitro* invasion inhibitory studies (Kinyanjui et al., 2007; Wright et al., 2014) which observed residues in or near basigin binding sites, including residues A205 and F209, are involved in interactions with the heavy chain of murine-derived monoclonal antibody (9AD4 and QA1) (Wright et al., 2014) or vaccine-induced antibodies (R5.004 and R5.016). Previous studies (Plenderleith et al., 2018) have hypothesized that RH5 residue S197Y is involved in the recent divergence of *P. falciparum* from its gorilla-infecting precursors such as *P. praefalciparum* and *P. adleri*, where 197Y can also be found (Naung et al., 2022). Polymorphic amino acid residues Y147H and H148D, which are in close proximity to the P113 binding region (Wright et al., 2014; Alanine et al., 2019) may also form epitopes. Precise spatial coordinates were not available for these residues on Cryo-EM structure because of the proximity to the N-terminal disordered region from residue 1 to 140 (Wong et al., 2019).

The RH5 – basigin interaction is essential for invasion and universally required by all strains of *P. falciparum* for human red-blood cell invasion (Hayton et al., 2008; Crosnier et al., 2011). Indeed, balancing selection found near the basigin binding sites of RH5 suggests that the basigin binding site is under diversifying selective pressure, which may be due to immune escape. As evidence that polymorphisms in the basigin binding site are critical for antibody recognition, a serine (S) to tyrosine (Y) mutation at residue 197 reduced the binding affinity of a monoclonal antibody *in vitro (* Alanine et al., 2019). Further *in vitro* parasite invasion or growth experiments investigating whether the children acquire strain-specific functional antibodies would be useful and could validate the importance of allele changes in subsequent infections.

Given the evidence of balancing selection at the parasite population level, it is surprising that RH5 polymorphism was not associated with a higher turnover rate within the children in this study cohort. This finding contrasts with other leading blood-stage vaccine antigens, such as AMA1 and CSP, which are diverse (Takala et al., 2007; Takala et al., 2009; Ouattara et al., 2013; Naung et al., 2022) and for which vaccine efficacy was constrained by vaccine escape by parasites carrying non-vaccine like alleles (Takala et al., 2009; Thera et al., 2011; Ouattara et al., 2013; Neafsey et al., 2015). Even though many of the young children in this cohort study (1-3 yrs old) with limited life-time exposure showed evidence of protection against severe malaria (Tessema et al., 2019), they have not yet acquired high levels of immunity against uncomplicated, clinical episodes of *P. falciparum* malaria. Therefore, immune selection may be undetectable in this cohort because it requires high titres of diverse antibodies protecting against multiple parasite strains carrying different RH5 alleles. Our ability to detect immune escape in these young children may therefore be limited, since they may be just as likely to experience symptoms from a different strain as any child experiencing any infection in this age group, warranting a repeat of this study in older cohorts who have more complex antibody repertoires. However, these older children will also have fewer clinical episodes to genotype. The haplotype network also supports the presence of balancing selection with several moderate frequency haplotypes, and there is an equal distribution of major haplotypes between asymptomatic and symptomatic infections suggesting there is no association of certain haplotypes with clinical symptoms. On the other hand, older children have fewer episodes of clinical malaria due to acquired immunity, making this analysis more challenging to perform, albeit a similar analysis comparing any infection irrespective of symptoms is feasible and relevant for hyper-immune older children (Marsh, 1992; Doolan et al., 2009). In addition, we could measure the association with higher parasitemia level, rather than clinical symptoms to account for the fact that older children may have high parasitemia but no symptoms due to antibodies against virulence determinants such as PfEMP1 (59,60). Thus, the turnover of alleles driven by immune escape of antigenically distinct alleles may only result in a symptomatic episode in older immune children if the antigenic overlap is minimal (Shah et al., 2021).

A lack of immune selection detected within these children may also be explained by the slow and limited acquisition of naturally acquired immunity to this specific antigen. Unlike AMA1 (Akpogheneta et al., 2008; Crompton et al., 2010), natural immunity may acquire slowly for antigens such as RH5, that are possibly hidden within the merozoite apical end during tight junction formation during the course of infection (Wong et al., 2019). Low immunogenicity of the RH5 complex in natural infections supports this possibility (Tran et al., 2014). Nevertheless, *in vitro* evidence of strain-transcending immunity suggests promising protective efficacy against parasite replication (Minassian et al., 2021; Singh et al., 2021; Willcox et al., 2021).

Another possible explanation for the observed pattern of balancing selection is variability in the host receptor basigin. While basigin polymorphisms contribute to the ‘OK’ blood group (Crosnier et al., 2011), to our knowledge, no association of these blood groups with malaria has been described. The PNG human population exhibits a number of polymorphisms associated with malaria protection such as Gerbich negativity, alpha thalassemia, Duffy antigen (FYA) and Southeast Asia Ovalocytosis, which are the result of mutations in erythrocyte membrane proteins (O’Donnell et al., 1998; Zimmerman et al., 1999; Patel et al., 2001; Goheen et al., 2017). BSG however has not been analyzed in PNG populations. Any balancing selection at BSG could explain the patterns observed in RH5 and is a priority for further investigation. This finding could also have implications for vaccine efficacy because inclusion of only one allele may lead to expansion of the non-vaccine allele and greater malaria risk in individuals with a potentially more receptive host basigin variant.

In conclusion, population sequencing of *rh5* genes in *P. falciparum* isolates sampled from young PNG children has demonstrated significant balancing selection at the basigin binding interface, however immune escape, based on the association of polymorphisms with clinical symptoms, was not observed. Due to the young age and therefore limited immunity of the children enrolled in the cohort, polymorphisms between sequential infections within the children, were just as likely as any two infections among children, and thus immune escape was not detected. While this does not rule out immune selection altogether, the evidence of balancing selection among rh5 gene sequences in the parasite population suggests as yet unknown selective pressures that may limit RH5 vaccine efficacy in this population. Development of RH5 vaccines should consider the inclusion of variants of RH5 that are representative of the parasite population, especially when the vaccine (3D7) haplotype is very low.

## Data availability statement

The datasets presented in this study can be found in online repositories. The names of the repository/repositories and accession number(s) can be found below: https://www.ncbi.nlm.nih.gov/genbank/, MT414033-MT414709.

## Ethics statement

The studies involving humans were approved by Medical Research Advisory Council of PNG No. 07/11; Walter and Eliza Hall Institute Human Research Ethics Committee No. 07/07; Deakin University Human Research Ethics Committee No. 2023-219. The studies were conducted in accordance with the local legislation and institutional requirements. Written informed consent for participation in this study was provided by the participants’ legal guardians/next of kin.

## Author contributions

MN: Data curation, Formal analysis, Investigation, Methodology, Software, Validation, Writing – original draft, Writing – review & editing. EM: Writing – review & editing, Data curation, Formal analysis, Investigation, Software. WW: Investigation, Methodology, Writing – review & editing. ZR: Investigation, Methodology, Resources, Writing – review & editing. DU: Investigation, Methodology, Resources, Writing – review & editing. AG: Software, Writing – review & editing. SH: Methodology, Resources, Supervision, Writing – review & editing. AC: Resources, Supervision, Writing – review & editing. EL: Resources, Writing – review & editing. BK: Resources, Writing – review & editing. ML: Resources, Writing – review & editing. IM: Conceptualization, Funding acquisition, Investigation, Methodology, Project administration, Resources, Supervision, Writing – review & editing. AB: Conceptualization, Funding acquisition, Investigation, Methodology, Project administration, Resources, Supervision, Visualization, Writing – review & editing.
