# Supplementary material for: Reticulocyte Binding Protein Homologue 5 is a target of balancing selection in the Plasmodium falciparum population of Papua New Guinea
Source: Front Parasitol. 2023 Dec 22;2:1288867. doi: 10.3389/fpara.2023.1288867 (PMC11731791; doi:10.3389/fpara.2023.1288867)
Supplement: Supplementary file 1 [file DataSheet_1.docx]

**Supplementary Material**

**Reticulocyte Binding Protein Homologue 5 is a target of balancing selection in the *Plasmodium falciparum* population of Papua New Guinea**

Myo T. Naung^1,2,3,4^, Elijah Martin^1^, Wilson Wong^1,2^, Zahra Razook^1,2,3,4^, Digjaya Utama^1,2^, Andrew J. Guy^5^, Shannon Takala-Harrison^6^, Alan F. Cowman^1,2^, Enmoore Lin^7^, Benson Kiniboro^7^, Moses Laman^7^, Ivo Mueller^1,2,8^ and Alyssa E. Barry^1,2,3,4^

1. Population Health and Immunity Division, Walter and Eliza Hall Institute of Medical Research, Parkville, Victoria, Australia

2. Department of Medical Biology, University of Melbourne, Carlton, Victoria, Australia

3. Centre for Innovation in Infectious Diseases and Immunology Research (CIIDIR), Institute of Mental and Physical Health and Clinical Translation (IMPACT) and School of Medicine, Deakin University, Geelong, Victoria, Australia

4. Disease Elimination and Maternal and Child Health, Burnet Institute, Melbourne, Victoria, Australia

5. Bioscience and Food Technology, RMIT University, Melbourne, Victoria, Australia

6. Center for Vaccine Development and Global Health, University of Maryland School of Medicine, Baltimore, Maryland, United States of America

7. Vector Borne Diseases Unit, Papua New Guinea Institute of Medical Research, Madang, Papua New Guinesa

8. Department of Parasites and Insect Vectors, Pasteur Institute, Paris, France

***Correspondence:**

Alyssa E. Barry

a.barry@deakin.edu.au

***Table S1:*** *Demographic information of the study participants*[1]

| Age | 0.9 to 3.2 years |
| --- | --- |
| *P. falciparum* infections | 2.56 episodes per child per year |
| mean molFOI* per year | 5.5 |
| Follow-up period | 16 months |

*molFOI was based on Mueller *et al.* (2012)[2], defined as the number of new *P. falciparum* clones are acquired over a specific unit of time.


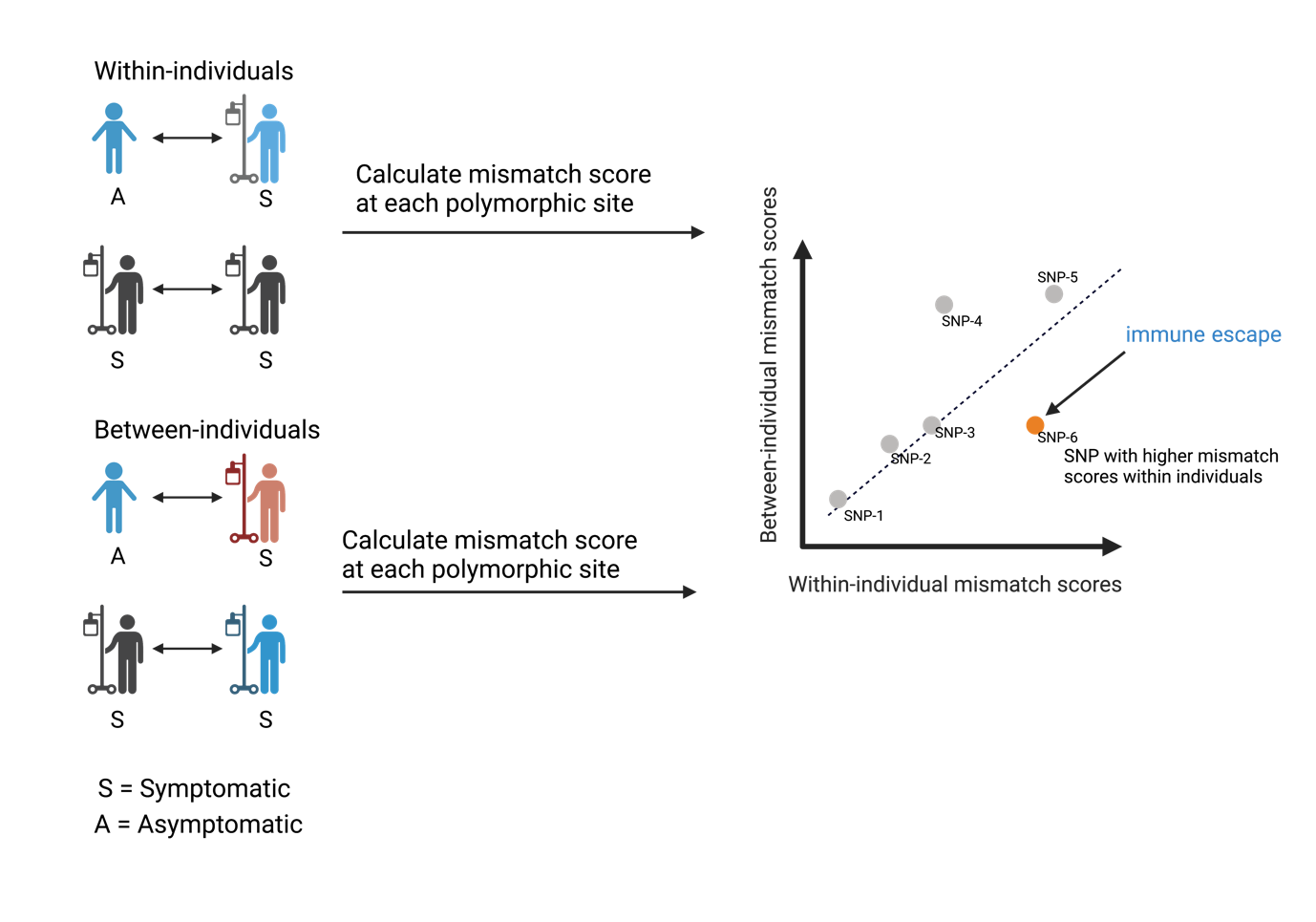


***Supplementary Figure S1.*** *Diagram of analysis to identify immune escape polymorphic sites within RH5 antigen.* *The analytical method focused on determining differences in the proportion of mismatched alleles within-host and the between-host at each polymorphic site. Between-hosts were used to adjust the random background mutation rate. Polymorphic sites associated with immune escape will have higher mismatch scores within host than between host. Permutation was used to test the null hypothesis.*

| 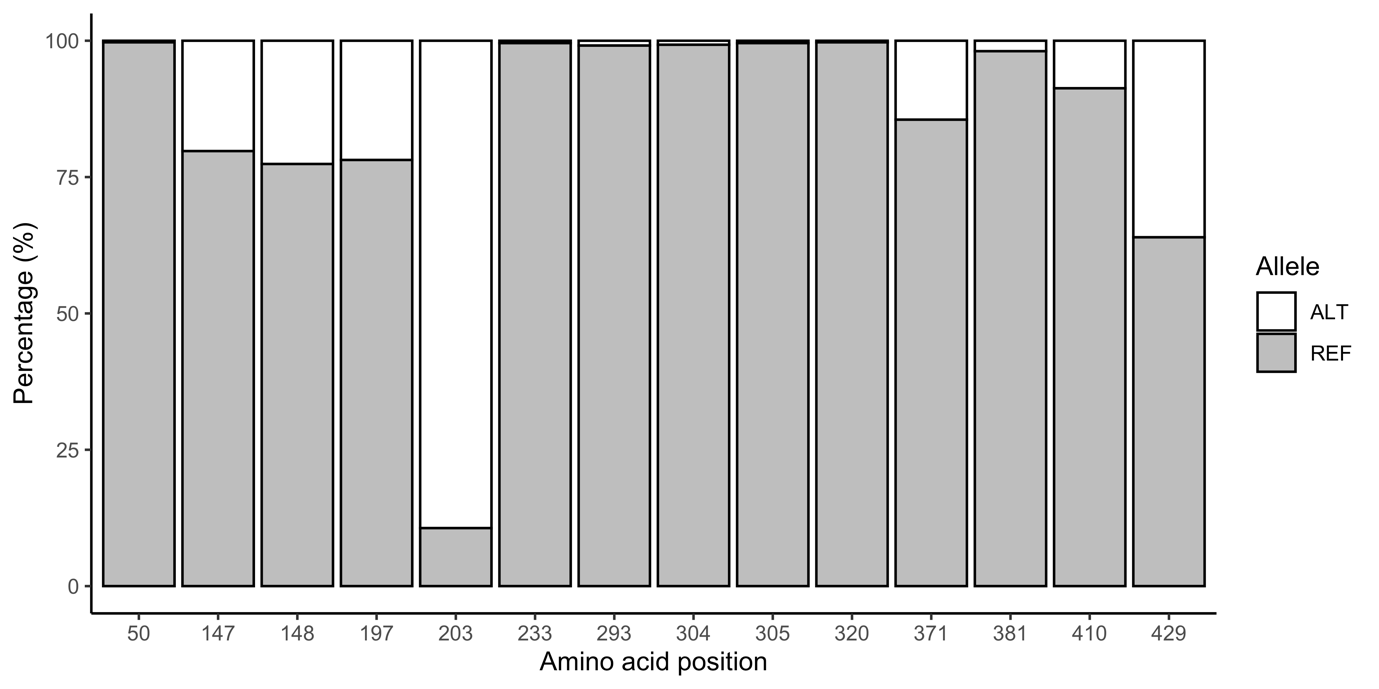 |
| --- |

***Fig S2.*** *RH5 amino acid changes and frequency of polymorphic alleles for RH5. The reference 3D7 allele (REF) frequency is gray, and the non-reference allele (ALT) is represented in maroon colors.*

**References**

1. Lin E, Kiniboro B, Gray L, Dobbie S, Robinson L, Laumaea A, et al. Differential Patterns of Infection and Disease with *P. falciparum* and *P. vivax* in Young Papua New Guinean Children. Ng LFP, editor. PLoS ONE. 2010;5: e9047. doi:10.1371/journal.pone.0009047

2. Mueller I, Schoepflin S, Smith TA, Benton KL, Bretscher MT, Lin E, et al. Force of infection is key to understanding the epidemiology of *Plasmodium falciparum* malaria in Papua New Guinean children. PNAS. 2012;109: 10030–10035. doi:10.1073/pnas.1200841109.
